# Job satisfaction among physiotherapists in Saudi Arabia: does the leadership style matter?

**DOI:** 10.1186/s12913-018-3184-9

**Published:** 2018-06-07

**Authors:** Othman Y. Alkassabi, Hana Al-Sobayel, Einas S. Al-Eisa, Syamala Buragadda, Ahmad H. Alghadir, Amir Iqbal

**Affiliations:** 1 Prince Sultan Bin Abdulaziz Humanitarian City, Riyadh, Saudi Arabia; 2 Department of Rehabilitation Sciences, College of Applied Medical Sciences, King Saud University, Riyadh, Saudi Arabia; 3 Rehabilitation Research Chair, College of Applied Medical Sciences, King Saud University, Riyadh, Saudi Arabia

**Keywords:** Applied psychology, Health management, Physiotherapy leadership

## Abstract

**Background:**

Research has shown high rates of stress and dissatisfaction among allied health professionals, including physiotherapists, having an adverse impact on workforce retention rates. This study aimed to examine the job satisfaction and influential factors among physiotherapists working in private and government hospitals of Saudi Arabia with a focus on leadership style.

**Methods:**

This was a cross-sectional observational study conducted among sixty-nine licensed physical therapists working in various health care settings in Riyadh. The Job Satisfaction Survey questionnaire was used to measure job satisfaction, and the Multifactor Leadership Questionnaire was used to measure perceptions of leadership style. Other data including demographic and work-related information were collected. Chi-square and Pearson’s correlation analysis were used to establish correlation among the variables.

**Results:**

The respondents from government and private hospitals showed non-significant differences (*p* > 0.05) among them on job satisfaction score, which was considered “ambivalent”. Some of the respondents “slightly disagreed” in terms of pay, promotion, fringe benefits, contingent reward, operating conditions, and communication; however, rest of them “slightly agreed” for immediate supervision, co-workers, and the nature of work. Job satisfaction correlated significantly with female gender (*p* < 0.05) and musculoskeletal subspecialty of physiotherapy (*p* < 0.05) however, correlated non-significantly with leadership style (*p* > 0.05).

**Conclusions:**

All the physiotherapists, whether working in government or private hospitals, were neither fully satisfied nor fully dissatisfied with their jobs. Female physiotherapists from musculoskeletal subspecialty of physiotherapy were more satisfied than male physiotherapists from other subspecialty of physiotherapy. Of course, leadership style does matter in the job satisfaction among physiotherapists in the kingdom of Saudi Arabia.

## Background

According to the World Health Organization (WHO), countries are facing challenges in building human capacity within health care systems. One of such challenges is the migration of health care workers from rural areas to urban ones within their countries; even from home countries to abroad looking for better working conditions, enumerations, and career opportunities. This migration results in increasingly unbalanced access to health care both within and between countries. To minimize these migrations, the key steps are to establish a sympathetic working and living environments and provide a prospect for professional growth for healthcare workers [1]. The major noticeable factor behind this migration is employee’s job dissatisfaction which further heavily influence the performance, quality of service and clinical outcomes of individuals and health care organizations too [2, 3]. Physiotherapy is a profession that can be highly stressful with personal and/or work-related factors adding to the pressure and influencing job satisfaction among physiotherapists [4].

Previously, researchers revealed that the different types of challenges such as working environment, expectation to get potential care from organization, progression in healthcare technologies, reformation of organization; provide important insight into the type of work characteristic those can result in to high rates of dissatisfaction, pressure exhaustion and retention of workforce among allied health professionals, including physiotherapists [2, 5]. Besides these types of work characteristics, there is lack of funding to allied health professionals also affected worryingly in addressing to the issues of increasing demands among allied health professionals [6]. In addition, the allied health professionals are limited to receive political gain along with financial resources in emphasizing the orientation of health care settings toward medical practitioners and medical solutions against the health issues [7, 8]. That’s why, these factors (work characteristics, financial and political influences) pushed the allied health professionals to face challenges of job-stress, job-dissatisfaction, low level of decision-making ability, disparate/reduction in workforce compared to physician and nurses [2]. The challenges of working in allied health become more intensified when associated with the deficiency of fundamental planning strategies to retain workforce and staff [9]. Furthermore, the worse carrier prospects, lesser recognition, lower salaries of allied health professionals compared to doctors, nurses and other medical staffs, highlighted as a significant source of dissatisfaction and less income [10].

The job demand and control are described as a psychosocial aspects of work environment in occupational health psychology [2]. The high job demand and low control create the job-stress/strain that play an important role in poor outcomes/turnovers [4, 11]. Job stress is reduced by organizational factors such as support and perceived importance/value. Perceived importance is related to the degree of autonomy, authority, recognition given to individuals and how the employees perceive themselves to be supported by the organization [12]. Moreover, intrinsic factor (i.e., an environment in line with one’s professional values) play as a dominating predictive factor for rehabilitation specialists including physiotherapists in terms of career satisfaction and desire to stay on the job; whereas extrinsic factor (i.e., competitive pay) have a weaker significance [13].

Job characteristics, such as autonomy or innovativeness, might not be relevant sources of well-being in all countries; their relevance may depend on the cultural and socioeconomic environment in which the employees work and live. In developed countries, workers may put more value on the intrinsic motivational factors emphasized by Western psychosocial approaches to employees whereas, in developing countries, the role of external motivational factors such as monetary rewards or a clean work environment may be more salient [14].

Information on job satisfaction among physiotherapists in the developing countries is limited. Fewer studies reported about the relation between job satisfaction and work-related factors such as pay, professional advancement and leadership quality/style [15, 16]. One of the study conducted in European country reported a significant, positive relationship between the perception of leadership quality and job satisfaction with leadership quality being one of the best predictors of job satisfaction among physiotherapists. The other factors that showed strong relationship with job satisfaction in this study included interpersonal relationships, income, opportunities for personal and professional growth, and professional advancement opportunities [15].

In Saudi Arabia, there is a lack of research on job satisfaction in allied health professionals, including physiotherapists. Recent research suggested a high level of job dissatisfaction among physiotherapists working in Saudi Arabia [16]. This study aimed to investigate job satisfaction and influential factors among physiotherapists in Saudi Arabia with special focus on leadership style, as this may help in recommending interventions in physiotherapy services that may lead to improvements in job satisfaction, staff retention, organizational performance and patient outcomes.

## Methods

### Study design and setting

This was a cross-sectional exploratory study design, used the self-reported questionnaires. Permission was sought from the authors of the Job Satisfaction Survey. Return of a completed survey was considered to represent consent from participants.

### Selection of participants

We approached a total of 123-physiotherapists working in 11 hospitals (6-public/government and 5-private) approved by ministry of health in Riyadh, Saudi Arabia using their e-mails obtained through our professional contacts only. Inclusion criteria were as follows; a licensed physiotherapist, having at least 2–3 years of current clinical experience, and a resident of Riyadh for at least 2 years.

### Procedure

A cross-sectional survey was conducted to explore the respondent’s job satisfaction and the associated factors with special focus on leadership style. Physiotherapists working in the selected hospitals were e-mailed with the attachment of the copy of a cover letter (explaining purpose of the study), a self-reported JSS and MLQ-SF questionnaires including demographic and work-related variable. A follow up e-mail was sent after 2-weeks of the initial dispatch to those participants who did not respond. All those physiotherapists return their completed questionnaire were included in the data analysis.

### Outcome measures

The main outcome measure was the JSS English version developed by Spector to assess the level of job satisfaction among physiotherapist [17]. This survey comprises 36-items grouped into 9-subscales (salaries, fringe benefits, recognition, promotion, communication, working conditions, nature of the job, supervision and coworkers), each subscale comprising 4-items. The six-point Likert scales used to measure the responses to each item ranging from strongly disagree = 1 to strongly agree = 6. The scores of 4-items of each subscale range from 4 to 24 and total JSS scores from 36 to 216. The mean score of 4-items of each subscale equal to 3 or less is interpreted as dissatisfaction, 4 or more is interpreted as satisfaction, and between 3 and 4 is interpreted as ambivalent. Like-wise, a total JSS score ranges between 36 and 108 is interpreted as dissatisfaction, 144 to 216 as satisfaction and 108 to 144 as ambivalent. Content and face validity, were established by a panel of experts consisting of management experts [16, 18, 19]. The internal consistency reliability of the job satisfaction questionnaire has approved in previous study with the employees of a group of hospitals by using the Cronbach’s alpha(α) coefficient ranged between ICC 0.82 to 0.90 [16, 18–21].

The MLQ contains 45-items that identify and measure key leadership and effectiveness behaviors. Each of the nine leadership components, along a full range of leadership styles, is measured by four highly inter-correlated items as low in correlation as possible with items of the other eight components, all rated using a 5-point scale [22]. The validity and reliability of this tool has already approved by various studies done on health care and allied health professionals including physiotherapists working in government and private hospitals [16, 18, 19, 21, 23].

### Operational definitions

#### Transformational leadership style

It was defined as transformational leadership is a leadership style that can stimulate and inspire followers to both achieve extraordinary outcomes and, in the process, develop their own leadership capacity. Transformational leaders are generally emotionally intelligent, energetic, enthusiastic, and passionate. Not only are these leaders concerned and involved in the process; they are also focused on helping every member of the group succeed as well [24–26].

#### Transactional leadership style

Transactional leadership, also known as managerial leadership, focuses on the role of supervision, organization, and group performance. Leaders who implement this style focus on specific tasks and use rewards and punishments to motivate followers [25]. One of the main advantages of this leadership style is that it creates clearly defined roles. Individual know what to do and what to receive in exchange for completing their tasks. It offers leaders to supervise well and motivate individual to perform well to get rewards [26].

#### Passive avoidance leadership style

In this leadership style, leader takes corrective action retrospectively against the significant and obvious problems only if occurs. In some cases, can be considered a transformational leader quality when the leader purposely aims to let followers learn from making mistakes. The major drawback of this leadership style is that the few of the followers start avoiding the decision, passes important decision making responsibility to subordinates, reluctance to express views on important or controversial issues [26].

### Data analysis

The responses to closed questions (e.g., demographic data) were tabulated, and descriptive statistics were obtained. The associations among job satisfaction and demographic and work-related factors were analyzed using chi-square, and the relationship between job satisfaction scores and leadership style was measured using Pearson’s correlation. All analyses were conducted using SPSS-21. Level of significance was set at *p* < 0.05.

## Results

### Descriptive results

A total of 69 out of 123-physiotherapists completed the questionnaire (response rate 56%). 36 (52%) worked in the public/government and 33 (48%) in the private sector. Their demographic and work-related characteristics are shown in Table 1.

**Table 1:** Demographic and work-related characteristics of the participants from government and private hospitals

Characteristic	Overall sample *N* = 69	Government hospitals *N* = 36	Private hospitals *N* = 33	*p*- values
Gender, *n* (%)				
Male	32 (46)	16 (44)	16 (48)	0.753
Female	36 (52)	20 (56)	16 (48)	
Unspecified	1 (1)	0	1 (3)	
Nationality, *n* (%)				
Saudi	33 (48)	26 (72)	7 (21)	0.000*
Non-Saudi	36 (52)	10 (28)	26 (79)	
Marital status, *n* (%)				
Single	28 (41)	16 (44)	12 (36)	0.542
Married	39 (57)	20 (56)	19 (58)	
Other	1 (1)	0	1 (3)	
Unspecified	1 (1)	0	1 (3)	
Highest academic degree, *n* (%)				
Bachelor of Science	29 (42)	18 (50)	11 (33)	0.950
Master of Science	15 (22)	10 (28)	5 (15)	
Doctor of Philosophy	2 (3)	2 (6)	0	
Other	1 (1)	1 (3)	0	
Unspecified	22 (32)	5 (14)	17 (52)	
Years of physiotherapy experience, mean (range)	10.5 (0.5–37.0)	9.9 (0.5**–**37.0)	11.2 (1.3**–**32.0)	0.027*
Years worked within the organization, mean (range)	6.5 (0.5**–**26.0)	6.1 (0.5**–**26.0)	7.0 (8.0**–**21.0)	0.387
Job subspecialty, *n* (%)				
Neurology	7 (10)	6 (17)	1 (3)	0.002*
Musculoskeletal	21 (30)	14 (39)	7 (21)	
Pediatrics	5 (7)	4 (11)	1 (3)	
Cardiopulmonary	2 (3)	0 (0)	2 (6)	
Lymphedema	2 (3)	2 (6)	0 (0)	
Women’s health	3 (4)	3 (8)	0 (0)	
General	7 (10)	3 (8)	4 (12)	
Unspecified	22 (32)	4 (11)	18 (55)	
Job position, *n* (%)				
Supervisor	11 (16)	7 (19)	4 (13)	0.005*
Senior therapist	20 (30)	15 (41)	5 (16)	
Junior therapist	29 (42)	10 (27)	19 (61)	
Other	1 (1)	0 (0)	1 (3)	
Unspecified	8 (12)	4 (11)	4 (13)	
Working hours per day, mean (range)	8.39 (4.5**–**14.0)	8.5 (4.5**–**10.0)	8.3 (6.0**–**14.0)	0.382
Number of patients seen per day, mean (range)	11 (1–18)	10 (1–18)	12 (2–14)	0.269

Chi-square analyses comparing between both of them

*significant value if *p* ≤ 0.05

There was a relatively even spread of men and women working in both sectors. Overall, the sample was nearly equally distributed between Saudi and non-Saudi nationals (*p* > 0.05); however, significantly more were Saudis in the public compared to the private sector (*p* = 0.00). The highest academic degree most frequently recorded was a bachelor’s degree. The area in which respondents worked differed significantly between sectors (*p* = 0.00). Twenty-one respondents (30%) worked mainly as musculoskeletal physiotherapists (job-subspecialty), and 22 (32%) did not specify their job-subspecialty. The rank of physiotherapists ranged from junior to supervisor. The distribution of different positions differed significantly between sectors (*p* = 0.00). Twenty-nine respondents (42%) worked mainly as junior physiotherapists, and 20 (30%) worked as senior physiotherapists. Respondents were similar between the public and private sectors in terms of years employed in their current position (*p* = 0.387), working hours (*p* = 0.382), and number of patients seen per day (*p* = 0.269), except regarding the number of years experienced (*p* = 0.027).

### Job satisfaction survey and leadership style

**Fig. 1:**
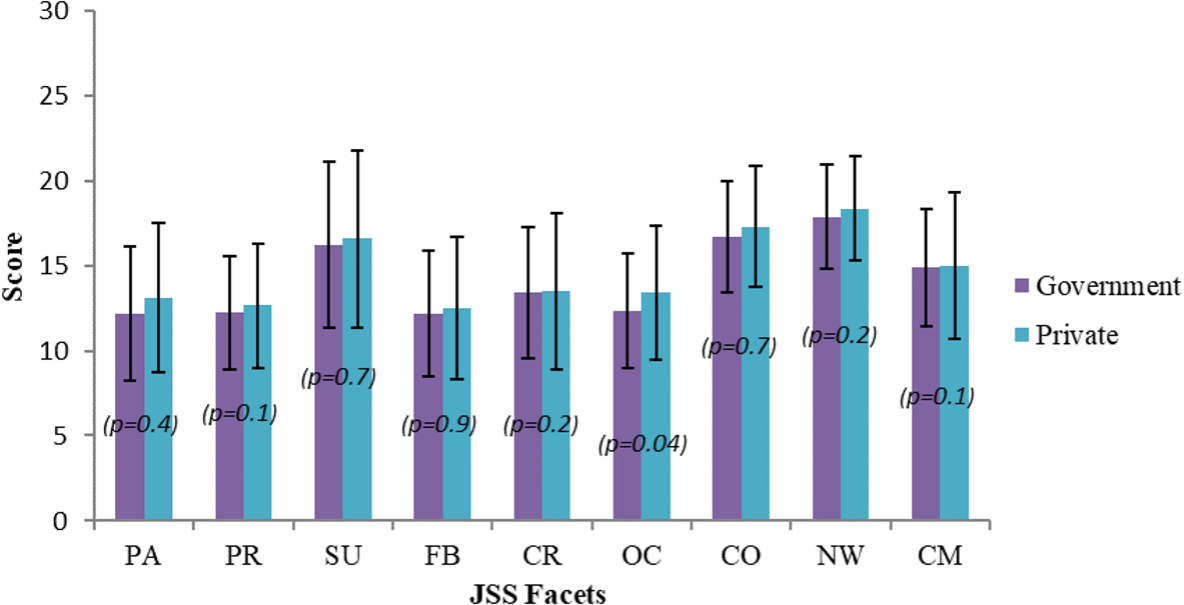
Comparison of mean scores for each subscales of the Job Satisfaction Survey between government sector and (*n* = 36) or private sector (*n* = 33). No significant difference in scores between government and private sectors (*p* > 0.05) except for OC (*p* < 0.05). JSS = Job Satisfaction Survey; PA = Pay, PR = Promotion, SU = Supervision, FB = Fringe Benefits, CR = Contingent Reward, OC = Operating Conditions, CO = Coworkers, NW = Nature of Work, CM = Communication

According to the JSS, the mean total score of JSS for participants in the current study was within the “ambivalent” category. Few participants scored on the lower end of the ambivalent range denoted as “slightly disagree or slightly dissatisfied” for the following subscales: pay (pay and remuneration), promotion (promotion opportunities), fringe benefits (monetary and nonmonetary fringe benefits), contingent reward (appreciation, recognition, and rewards for good work), operating conditions (OC; operating policies and procedures), and communication (communication within the organization). However, the rest participants scored on the upper end of ambivalent range denoted as “slightly agree or slightly satisfied” for the following subscales: supervision (immediate supervisor), co-workers (people you work with), and nature of work (job tasks themselves). No significant differences were seen between participants in the public and private sectors either for JSS total or subscale scores except for OC, as described in Fig. 1. The relation between job satisfaction scores and demographic or work-related factors was significant and positive only for the gender; more in female than male PTs (*p* = 0.019) and job-subspecialty; more in musculoskeletal physiotherapy than others subspecialties of physiotherapy (*p* = 0.038).

**Fig. 2:**
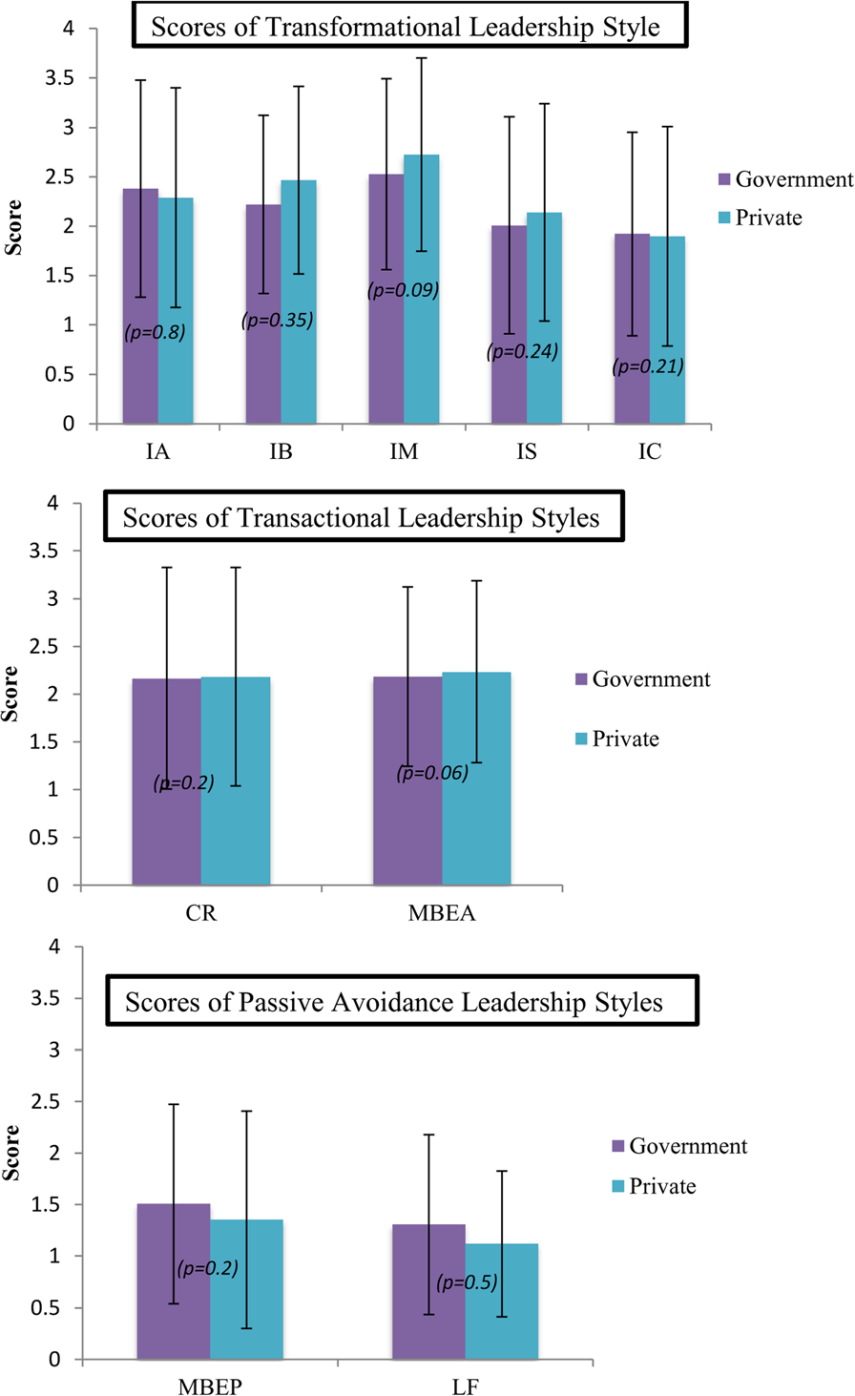
Comparison of scores of leadership style between government sector and (*n* = 36) or private sector (*n* = 33). No significant difference in scores between government and private sectors (*p* > 0.05). IA = Idealized Attributes, IB = Idealized Behaviors, IM = Inspirational Motivation, IS = Intellectual Stimulation, IC = Individual Consideration, CR = Contingent Reward, MBEA = Management by Exception (Active), MBEP = Management by Exception (Passive), LF = Laissez-Faire

As for leadership style, based on the participants’ perceptions of their managers, they showed more transformational and transactional leadership styles than passive avoidance style, as described in Fig. 2.

The analysis between job satisfaction and leadership style showed average to highly strong correlation among any of the leadership styles and total job satisfaction scores or job satisfaction facets, as described in Table 2.

**Table 2:** Correlation between job satisfaction facets/subscales and leadership styles

Leadership Styles		JSS Facets/subscales									
		PAY	PR	SU	FB	CR	OC	CO	NW	CM	JSS total
Transformational Leadership Style	IA	.975	.630	.550	.884	.949	.922	.707	.420	.859	.765*
	IB	.735	.602	.601	.747	.807	.761	.714	.486	.937	.979*
	IM	.706	.977	.386	.982	.840	.516	.991	.699	.815	.978*
	IS	.812	.414	.844	.863	.371	.841	.665	.985	.517	.664*
	IC	.837	.466	.689	.852	.874	.806	.721	.663	.309	.783*
Transactional Leadership Style	CR	.896	.701	.676	.893	.557	.436	.888	.743	.797	.804*
	MBEA	.397	.751	.980	.257	.589	.275	.748	.159	.624	.914*
Avoidance Style	MBEF	.975	.128	.902	.948	.396	.445	.415	.541	.711	.990*
	LF	.980	.543	.282	.887	.464	.727	.319	.924	.859	.596*
Outcomes	EE	.659	.910	.596	.759	.815	.403	.523	.633	.927	.568*
	EFF	.776	.431	.669	.985	.897	.938	.751	.790	.718	.957*
	SAT	.998	.647	.780	.873	.635	.441	.723	.774	.674	.981*

*PA* Pay, *PR* Promotion, *SU* Supervision, *FB* Fringe benefits, *CR* Contingent Reward, *OC* Operating Conditions, *CO* Co-workers, *NW* Nature of Work, *CM* Communication, *JSS* Job Satisfaction Survey, *IA* Idealized Attributes, *IB* Idealized Behaviors, *IM* Inspirational Motivation, *IS* Intellectual Stimulation, *IC* Individual Consideration, *CR* Contingent Reward, *MBEA* Management by Exception (Active), *MBEP* Management by Exception (Passive), *LF* Laissez-Faire, *EE* Extra Effort, *EFF* Effectiveness, *SAT* Satisfaction

*Significant at *p* ≤ 0.05

All dimensions of transformational leadership style showed moderate to highly strong correlation with JSS total scores as obtained for idealized attributes (*r* = 0.765), idealized behaviors (*r* = 0.979), inspirational motivation (*r* = 0.978), intellectual stimulation (*r* = 0.664), and individual consideration (*r* = 0.783). Likewise, all dimensions of transactional leadership style showed moderate to highly strong correlation with JSS total scores as obtained for contingent reward (*r* = 0.804) and active management-by-exception (*r* = 0.914). Similarly, all passive avoidance leadership style dimensions showed average to highly strong correlation with JSS total scores as obtained for passive management-by-exception (*r* = 0.990) and laissez-faire (*r* = 0.596). In addition, leadership style outcomes also showed average to highly strong correlation with JSS total scores as obtained for effective effort (*r* = 0.568), effectiveness (*r* = 0.957), and satisfaction (*r* = 0.981).

Despite presenting moderate to strong correlation between all leadership style dimensions and job satisfaction facets, fewer showed very poor to poor correlation for the same, such as between IA and NW (*r* = 0.420), IB and NW (*r* = 0.486), IM and SU (*r* = 0.386), IS and PR (*r* = 0.414), IS and CR (*r* = 0.371), IC and PR (*r* = 0.466), IC and CM (*r* = 0.309), CR and OC (*r* = 0.436), MBEA and FB (*r* = 0.257), MBEA and OC (*r* = 0.275), MBEA and NW (*r* = 0.159), MBEP and PR (*r* = 0.128), MBEP and CR (*r* = 0.396), MBEP and OC (*r* = 0.445), MBEP and CO (*r* = 0.415), LF and SU (*r* = 0.282), LF and CR (*r* = 0.464), LF and CO (*r* = 0.319), EE and OC (*r* = 0.403), EFF and PR (*r* = 0.431), and SAT and OC (*r* = 0.441).

## Discussion

This study aimed to investigate the job satisfaction and influential factors of physiotherapists working in Riyadh, Saudi Arabia. Overall, job satisfaction was scored as ambivalent with job satisfaction significantly associated with gender and job-subspecialty.

The current study showed that the participants, whether working in the public/government or private sector, were ambivalent with respect to satisfaction with their jobs. The issues that contributed highly to the total scores of job satisfaction included pay, promotion, fringe benefits, contingent reward, OC, and communication. Thus, the participants were undecided regarding satisfaction with these aspects of their jobs. On the other hand, they showed slightly higher scores, representing greater job satisfaction, in other aspects of their jobs, including their immediate supervisors, co-workers, and the nature of the work. This contrasts with the study by Eker et al. [15], in which almost 54% (out of 198) of participating Turkish physiotherapists were dissatisfied with their jobs specially in the area of salary and advancement, which they suggested was due to leadership quality. They reported a significant, positive relationship between the perception of leadership quality and job satisfaction and declared the leadership quality is one of the best predictors of job satisfaction [15].

The findings of our study is in line with other studies, indicate that to increase physiotherapists’ job satisfaction, attention is required to improve salaries, benefits, recognition, and communication and promotion schemes [15–17, 22, 27–30]. These factors may be related to job autonomy, which is shown to be highly correlated with job satisfaction [14]. It should be noted that these results reflect the participants’ perceptions of these aspects of their jobs, which may be affected by intrinsic factors such as self-determination and perceived competence [27]. Therefore, any measures to improve job satisfaction using extrinsic factors should consider the intrinsic factors and how individuals perceive their self-esteem, generalized self-efficacy, locus of control and emotional stability [28]. Understanding these factors is important for managers to improve workers’ job satisfaction, well-being and work performance [29].

The current study showed no significant association between job satisfaction and any personal or work characteristics other than gender (female>male) and job-subspecialty (musculoskeletal physiotherapy>others subspecialty of physiotherapy). While one must be careful in drawing conclusions from these results due to the small sample size, they suggest possible gender difference in perceptions of job satisfaction. However, in line with current study previous study reported that, the female PTs reflected higher levels of job satisfaction than male PTs that indicate female PTs may differ from male PTs in expectations and priorities with respect to their jobs [16]. Additionally, women have various life priorities, issues and challenges than men, however the success in their career solely depend on the role of maintaining the balance between work responsibilities, marital integrity, and parental care [31].

On the other side, one study reported that male PT students exhibited higher expectations and goals at the beginning of their professional career, such as owning a private practice or becoming a manager or administrator, than female students [30]. Moreover, it is difficult for the men to quit their jobs/works even they become unhappy with it just because of maintaining their status and roles in their society, particularly in a male dominating society like Saudi Arabia where men accept the challenges and responsibilities of bread and butter for their families [16]. In contrast, gender differences with job satisfaction may be related to differences between genders in their beliefs regarding the control of one’s job. It was suggested that women at work show more external locus of control, which predicts lower job satisfaction compared to men [32]. Similarly, in a previous study, researchers found that only 48% of women and 50% of men were satisfied with their job in terms salary, professional growth and quality of leadership [15]. In the current study, the gender difference may also be related to cultural background and differing work situations between men and women and requires further investigation.

Another factor that showed a significant association with job satisfaction was job-subspecialty (musculoskeletal physiotherapy). Different job-subspecialties have different job demands, which lead to different job stressors [2]. Previous study had declared the intrinsic factors like professional’s working environment as per the requirements of the job-subspecialty as a dominating predictive factor for rehabilitation specialists (including physiotherapists) in terms of career satisfaction and desire to stay on the job; extrinsic factors like competitive pay had a weaker significance [13].

In a recently published study, researchers conducted a survey in Western Australia; aimed to capture factors contributing to the job satisfaction of musculoskeletal physiotherapists working in private practice across different career stages (new graduates, graduates, postgraduates, and owners). They found the owner group of musculoskeletal physiotherapists were significantly more satisfied with their job than rest group of musculoskeletal physiotherapists [33]. Furthermore, in another study a self-administered online survey used to conduct among 157 Notre Dame physiotherapy graduates using a job satisfaction rating scale. Their aim was to identify the relationship between job satisfaction and physiotherapists working in different subspecialty of physiotherapy (i.e., musculoskeletal, neurology, cardiorespiratory, and pediatrics physiotherapy). Their survey reported that the job satisfaction is significantly correlated with the physiotherapists practicing across different subspecialty such as pediatric (41.6%), musculoskeletal (39.2%), Geriatric care (37.4%) Neurology (36.8%) and cardiorespiratory (31.7%) physiotherapy [34]. This could explain the differences in job satisfaction between the various job-subspecialties.

Although there was lack of evidence to identify and develop the best leadership, it was hypothesized that leadership interventions are complex and require interaction between the employees, employers and leaders [35]. One of the main factors emphasized in the current study was leadership style. It was predicted that leadership style may be correlated with job satisfaction. In conformity, the results revealed average to strong positive correlation between the two variables. The aspect of leadership was shown to be more positive in both job satisfaction and leadership style surveys. This is in accordance with the literature, which suggested leadership as a predictor of job satisfaction [15, 20].

Participants were satisfied with their supervisors; they also perceived their leadership style to be more transformational or transactional than passive/avoidant. The agreement between these aspects of leadership measured by two different scales may indicate that leadership within this context is more towards the positive end of the spectrum. However, further understanding of this phenomenon is required as reports in the literature highlighted the importance of leadership and leadership styles for job satisfaction and that, by using appropriate leadership styles, managers can affect employee job satisfaction, commitment and productivity [20, 21, 35].

The main limitation of this study was the small sample size, limited to fewer participants out of a large number of physiotherapists working in the Riyadh region only. The response rate was low (46%) with respect to the number of e-mails sent to a total of 123-physiotherapists only as we could approach through our professional contacts only. Additionally, the length of the survey (JSS and MLQ) was large that could produce very low response as it could be difficult for therapists to complete within their busy schedules. In the future, we suggest larger sample size, use of paper instead of online surveys for a higher response rate, use of professional and in-service education meetings, or linking completion of these surveys to required work form updates within each institution.

## Conclusions

None of the physiotherapists, whether working in government or private hospitals, was fully satisfied with his/her job. Some “slightly disagreed” in terms of pay, promotion, fringe benefits, contingent reward, OC, and communication; however, the rest “slightly agreed” for immediate supervision, co-workers, and the nature of the work. Overall job satisfaction was significantly associated with demographic factors like gender (female>male) and job-subspecialty (musculoskeletal physiotherapy>other PT-subspecialties). Furthermore, the participants’ perception of the leadership styles of their managers or supervisors showed more transformational and transactional than passive avoidance style. There was positive correlation between JSS facets, JSS total and any of leadership style related to the success of the group. The participants perceived their leaders to be motivating and interacting at different levels of the organization and were satisfied with their leaders’ methods of working with others. This study will raise the awareness on the importance of job satisfaction among physiotherapist in Saudi Arabia. Another area that requires further focus to investigate the impact of job satisfaction among physiotherapists working in Saudi Arabia on individual performance, well-being, and patient outcomes.

## Abbreviations

CM: Communications
CO: Co-workers
CR: Contingent rewards
EE: Extra effort
EFF: Effectiveness
FB: Fringe benefits
IA: Idealized attributes
IB: Idealized behavior
IC: Individual considerations
IM: Inspirational motivations
IS: Intellectual stimulations
JSS: Job Satisfaction Survey
LF: Laissez-faire
MBEA: Management by exception (active)
MBEP: Management by exception (passive)
MLQ-SF: Multifactor Leadership Questionnaire Short-Form
NW: Nature of works
OC: Operating conditions
PR: Promotions
SAT: Satisfaction
SU: Supervision
